# Seroprevalence of *Toxoplasma gondii* infection in domestic cats in central China

**DOI:** 10.1051/parasite/2017010

**Published:** 2017-03-21

**Authors:** Shuai Wang, Yonghua Zhou, Jingyuan Niu, Qing Xie, Tingwei Xiao, Yunchao Chen, Han Li, Chenchen Ma, Haizhu Zhang, Shiguo Liu, Zhenchao Zhang

**Affiliations:** 1 School of Basic Medical Sciences, Xinxiang Medical University Xinxiang Henan 453003 PR China; 2 Jiangsu Institute of Parasitic Diseases, Key Laboratory on Technology for Parasitic Disease Prevention and Control, Ministry of Health, Jiangsu Provincial Key Laboratory on Molecular Biology of Parasites Wuxi Jiangsu 214064 PR China

**Keywords:** *Toxoplasma gondii*, Cat, Seroprevalence, ELISA, Central China

## Abstract

To investigate the seroprevalence of *Toxoplasma gondii* infection in domestic cats in central China, 843 serum samples were collected in Henan province between March 2015 and May 2016 and tested for IgG antibodies against *T. gondii* using the enzyme-linked immunosorbent assay (ELISA). The overall seroprevalence of *T. gondii* was 21% (178/843). No significant difference was observed based on the sex of cats (*p* > 0.05). Significantly higher seroprevalence (*p* < 0.05) was observed in mixed-breed cats (24%) compared to purebred cats (17%). Seroprevalence in rural cats (29%) was significantly higher (*p* < 0.01) than in urban cats (16%), and increased significantly (*p* < 0.01) with age. These results showed that *T. gondii* was highly prevalent in domestic cats in Henan province, central China, which might have important implications for public health.

## Abstract (fr)

Pour étudier la séroprévalence de l’infection par *Toxoplasma gondii* chez les chats domestiques en Chine centrale, 843 échantillons de sérum ont été prélevés dans la province du Henan entre mars 2015 et mai 2016 et testés pour l’anticorps IgG contre *T. gondii* par méthode immuno-enzymatique (ELISA). La séroprévalence globale de *T. gondii* était de 21 % (178/843). Aucune différence significative n’a été observée selon le sexe des chats (*p* > 0.05). Une séroprévalence significativement plus élevée (*p* < 0.05) a été observée chez les chats de race mixte (24 %) par rapport aux chats de race pure (17 %). La séroprévalence chez les chats en milieu rural (29 %) était significativement (*p* < 0.01) plus élevée que chez les chats urbains (16 %), et augmentait de façon significative (*p* < 0.01) avec l’âge. Ces résultats ont montré que *T. gondii* était très répandu chez les chats domestiques dans la province du Henan, en Chine centrale, ce qui pourrait avoir des implications importantes pour la santé publique.

## Introduction

*Toxoplasma gondii* is an obligate intracellular protozoan parasite that has a worldwide distribution and infects a wide range of warm-blooded vertebrates, including humans [3]. The ingestion of undercooked meat containing tissue cysts and the ingestion of food or drink contaminated with sporulated *T. gondii* oocysts are important modes of transmission of *T. gondii*. As definitive hosts of this parasite, cats play an important role in the life cycle of *T. gondii*, facilitating the genetic recombination between strains, as well as environmental contamination [12]. Antibodies to *T. gondii* have been reported in cats worldwide and seroprevalence in cats was recently summarized [2, 4, 8, 9]. The objective of the present survey was to determine the seroprevalence of *T. gondii* in domestic cats in Henan province, central China, and to evaluate the main associated risk factors related to exposure to *T. gondii* in this region [6, 14].

## Materials and methods

### Ethics statement

The study was reviewed and approved by the Ethics Review Committee of the Xinxiang Medical University (Reference No. 2015016).

### Study site

The study was conducted in Henan province in the central part of mainland China, covering an area of 167, 000 km^2^ and having a population of approximately 106 million. Its geographical position is at east longitude 110°21′–116°39′ and at north latitude 31°23′–36°22′. The Yellow River passes through central Henan. The area has a continental monsoon climate, with four distinctive seasons. The average annual temperature is 12.1–15.7 °C, with a mean annual rainfall of 532.5–1380.6 mm. There are 17 provincial cities distributed in Henan province, with the city of Zhengzhou as its capital. Five cities, including Anyang (35°13′–36°22′ N, 113°37′–114°58′ E), Sanmenxia (33°31′–35°05′ N, 110°21′–112°01′ E), Zhengzhou (34°16′–34°58′ N, 112°42′–114°13′ E), Xinyang (31°46′–31°52′ N, 114°01′–114°06′ E), and Shangqiu (33°43′–34°52′ N, 114°49′–116°39′ E), located in the northern, western, central, southern, and eastern parts of Henan province, were selected for sample collections.

### Sample collection

A total of 843 blood samples of domestic cats were collected from these five cities in Henan province between March 2015 and May 2016. Cat owners were asked for details of the animal’s age, sex, source, and breed using a structured questionnaire. Blood samples were centrifuged and sera were recovered and transferred to 1.5 mL Eppendorf tubes. All the sera were then stored at −80 °C until tested for anti-*T. gondii* antibodies.

### Determination of antibodies to *T. gondii*

Antibodies to *T. gondii* were determined using the commercial *T. gondii* IgG ELISA (enzyme-linked immunosorbent assay) Kit (Combined Company, Shenzhen, Guangdong Province, China) according to the manufacturer’s instructions [5, 13]. The manufacturer’s test report showed that the sensitivity and specificity of this ELISA kit were 95% and 100%, respectively. Positive and negative control sera were provided in the kit. Briefly, the *T. gondii* specific antigen was used to coat a 96-well ELISA plate. After incubation of the diluted serum sample (1:100) in the test well and subsequent washing, a conjugate was added. The plate was washed again and the chromogenic enzyme substrate was added. Finally, the optical density (OD) values of each well were measured using an ELISA plate reader (Multiskan MK3; Thermo Scientific, Waltham, MA, USA) at 450 nm.

A relative rate percent (IRPC) value was obtained using the following formula:

$$\mathrm{IRPC}=\frac{\mathrm{OD}450\;\left(\mathrm{sample}\right)-\mathrm{mean}\;\mathrm{OD}450\;(\mathrm{blank}\;\mathrm{control})}{\mathrm{mean}\;\mathrm{OD}450\;\left(\mathrm{negative}\;\mathrm{control}\right)-\mathrm{mean}\;\mathrm{OD}450\;(\mathrm{blank}\;\mathrm{control})}$$

The sera were considered negative to *T. gondii* if IRPC < 2.1 and positive if IRPC > 2.1.

### Statistical analysis

Differences in *T. gondii* prevalence for different variables such as age, breed, and sex were analyzed using a chi square test. Statistical analysis was performed using SPSS 20 software for Windows (SPSS Inc., Chicago, Illinois, USA). The differences were considered statistically significant if *p* < 0.05.

## Results

Antibodies to T*. gondii* were detected in 21.12% (178/843) of cats (Table 1). Seropositivity percentages from different cities were: 21.84% of 174 from Anyang, 14.56% of 158 from Sanmenxia, 30.54% of 167 from Zhengzhou, 17.68% of 181 from Xinyang, and 20.86% of 163 from Shangqiu.

**Table 1.** Seroprevalence of *Toxoplasma gondii* infection in domestic cats in Henan province, central China.

Variable	No. examined	No. of positive	Prevalence (%)	95% CI	χ^2^	*p*-Value
Region						
Anyang	174	38	21.84	15.70–27.98	14.327	<0.001
Sanmenxia	158	23	14.56	9.06–20.06		
Zhengzhou	167	51	30.54	23.55–37.52		
Xinyang	181	32	17.68	12.12–23.24		
Shangqiu	163	34	20.86	14.62–27.10		
Gender						
Male	416	92	22.16	18.13–26.10	0.493	0.482
Female	427	86	20.14	16.34–23.94		
Breed						
Purebred	324	54	16.67	12.61–20.72	6.252	0.012
Mixed-breed	519	124	23.89	20.22–27.56		
Area						
Urban	532	87	16.35	13.21–19.50	19.630	<0.001
Rural	311	91	29.26	24.20–34.32		
Age (years)						
≤1	214	15	7.01	3.59–10.43	86.813	<0.001
1–3	276	41	14.86	10.66–19.05		
3–6	198	53	26.77	20.60–32.93		
≥6	155	69	44.52	36.69–52.34		
Total	843	178	21.12	18.36–23.87		

The seroprevalence of *T. gondii* was higher in males (22.16%, 92/416) than in females (20.14%, 86/427) but the difference was not significant (*p* > 0.05). The seroprevalence of *T. gondii* infection was 16.67% (54/324) in purebred cats and 23.89% (124/519) in mixed-breed cats, showing a significant difference by breed (*p* < 0.05). Moreover, significantly higher seroprevalence was found in rural cats (29.26%), compared to that of cats raised in urban area (16.35%) (*p* < 0.01).

The prevalence of *T. gondii* infection in cats increased significantly (*p* < 0.01) with the increase in age.

## Discussion

The present survey showed that *T. gondii* seroprevalence in cats in rural areas was significantly higher compared to urban cats (*p* < 0.01), which is consistent with reports by other authors [1, 10]. The difference in seroprevalence in urban and rural areas might be explained by differences in local reservoirs of the parasite, both in prey animals and in the environment, which serve as local infection sources for the cats. In Henan, domestic cats are usually kept outdoors in rural areas and often roam more freely with greater access to parasites. In addition, the number of rodents in rural areas is much higher than that in urban areas. Higher *T. gondii* seroprevalence found in cats in rural areas probably results from the carnivorous behavior of cats living outdoors and eating prey animals such as rodents and birds. The higher seropositivity with increasing age of the cats supports the hypothesis that most cats acquire *T. gondii* infection after weaning [1, 7, 13].

In order to protect public health, more measures should be taken. The proper disposal of cat litter, keeping cats indoors to minimize their acquisition of infection from prey or the environment, and reducing the feral cat population are recommended. Reducing close contact with cats and protecting the play areas of children might potentially reduce the oocyst burden [11]. What is more, children, pregnant women, and immunocompromised people should adhere to hygiene principles after contact with soil and cats. Given the high seroprevalence of *T. gondii* IgG antibodies in cats, performing a screening test and determination of the IgG antibody titer in high-risk populations such as pregnant women in Henan province, central China is recommended.

## Conflict of interest

The authors declare that they have no conflict of interest.

## References

[1] Ahmad N, Ahmed H, Irum S, Qayyum M. 2014 Seroprevalence of IgG and IgM antibodies and associated risk factors for toxoplasmosis in cats and dogs from sub-tropical arid parts of Pakistan. Tropical Biomedicine, 31(4), 777–784. 25776604

[2] Alvarado-Esquivel C, Romero-Salas D, Cruz-Romero A, Garcia-Vazquez Z, Peniche-Cardena A, Ibarra-Priego N, Ahuja-Aguirre C, Perez-de-Leon AA, Dubey JP. 2014 High prevalence of *Toxoplasma gondii* antibodies in dogs in Veracruz, Mexico. BMC Veterinary Research, 10, 191. 25134696 10.1186/s12917-014-0191-x PMC4236830

[3] Dubey JP, Jones JL. 2008 *Toxoplasma gondii* infection in humans and animals in the United States. International Journal for Parasitology, 38(11), 1257–1278. 18508057 10.1016/j.ijpara.2008.03.007

[4] Lopes AP, Santos H, Neto F, Rodrigues M, Kwok OC, Dubey JP, Cardoso L. 2011 Prevalence of antibodies to *Toxoplasma gondii* in dogs from northeastern Portugal. Journal of Parasitology, 97(3), 418–420. 21506866 10.1645/GE-2691.1

[5] Liu QX, Wang S, Wang LQ, Xing J, Gao WJ, Liu GF, Zhao B, Zhang HB, Gao LH. 2014 Seroprevalence of *Toxoplasma gondii* infection in dogs and cats in Zhenjiang City, Eastern China. Asian Pacific Journal of Tropical Biomedicine, 4(9), 725–728.

[6] Li F, Wang S-P, Wang C-J, He S-C, Wu X, Liu G-H. 2016 Seroprevalence of *Toxoplasma gondii* in goats in Hunan province, China. Parasite, 23, 44. 27762212 10.1051/parasite/2016053 PMC5075832

[7] Must K, Lassen B, Jokelainen P. 2015 Seroprevalence of and risk factors for *Toxoplasma gondii* infection in cats in Estonia. Vector-Borne and Zoonotic Diseases, 15(10), 597–601. 26394196 10.1089/vbz.2015.1809

[8] Machacova T, Bartova E, Sedlak K, Slezakova R, Budikova M, Piantedosi D, Veneziano V. 2016 Seroprevalence and risk factors of infections with *Neospora caninum* and *Toxoplasma gondii* in hunting dogs from Campania region, southern Italy. Folia Parasitologica, 63, 12. 10.14411/fp.2016.012 27189127

[9] Nguyen TT, Choe SE, Byun JW, Koh HB, Lee HS, Kang SW. 2012 Seroprevalence of *Toxoplasma gondii* and *Neospora caninum* in dogs from Korea. Acta Parasitologica, 57(1), 7–12. 22807008 10.2478/s11686-012-0010-0

[10] Oi M, Yoshikawa S, Maruyama S, Nogami S. 2015 Comparison of *Toxoplasma gondii* seroprevalence in shelter cats and dogs during 1999–2001 and 2009–2011 in Tokyo, Japan. PLoS One, 10(8), e0135956. 26284780 10.1371/journal.pone.0135956 PMC4540423

[11] Torrey EF, Yolken RH. 2013 *Toxoplasma* oocysts as a public health problem. Trends in Parasitology, 29(8), 380–384. 23849140 10.1016/j.pt.2013.06.001

[12] Vilares A, Gargate MJ, Ferreira I, Martins S, Julio C, Waap H, Angelo H, Gomes JP. 2014 Isolation and molecular characterization of *Toxoplasma gondii* isolated from pigeons and stray cats in Lisbon, Portugal. Veterinary Parasitology, 205(3–4), 506–511. 25195193 10.1016/j.vetpar.2014.08.006

[13] Zhang H, Zhou DH, Zhou P, Lun ZR, Chen XG, Lin RQ, Yuan ZG, Zhu XQ. 2009 Seroprevalence of *Toxoplasma gondii* infection in stray and household cats in Guangzhou, China. Zoonoses and Public Health, 56(9–10), 502–505. 19243564 10.1111/j.1863-2378.2008.01209.x

[14] Zhang N, Wang S, Wang D, Li C, Zhang Z, Yao Z, Li T, Xie Q, Liu S, Zhang H. 2016 Seroprevalence of *Toxoplasma gondii* infection and risk factors in domestic sheep in Henan province, central China. Parasite, 23, 53. 27882868 10.1051/parasite/2016064 PMC5134671

